# Complementing or Congruent? Desired Characteristics in a Friend and Romantic Partner in Autistic versus Typically Developing Male Adolescents

**DOI:** 10.1007/s10508-022-02444-y

**Published:** 2022-10-14

**Authors:** Linda P. Dekker, Esther J. M. van der Vegt, Anneke Louwerse, Kirsten Visser, Jan van der Ende, Athanasios Maras, Frank C. Verhulst, Kirstin Greaves-Lord

**Affiliations:** 1 Department of Psychology, Education and Child Studies, Erasmus University Rotterdam, Burg. Oudlaan 50, Room T13-24, 3000 DR Rotterdam, The Netherlands; 2 Department of Child and Adolescent Psychiatry/Psychology, Erasmus University MC/Sophia Children’s Hospital, Rotterdam, The Netherlands; 3 Yulius, Organization for Mental Health, Rotterdam, The Netherlands; 4 Youz Child and Adolescent Psychiatry, Team Sarr Autism Expertise Centre, Rotterdam, The Netherlands; 5 Child and Adolescent Mental Health Center, Mental Health Services, Capital Region of Denmark, Copenhagen, Denmark; 6 Faculty of Health and Medical Sciences, Department of Clinical Medicine, University of Copenhagen, Copenhagen, Denmark; 7 Autism Team Northern-Netherlands of Jonx, Department of (Youth) Mental Health and Autism, Lentis Psychiatric Institute, Groningen, The Netherlands; 8 Department of Clinical Psychology and Experimental Psychopathology, Faculty of Behavioral and Social Sciences, University of Groningen, Groningen, The Netherlands

**Keywords:** Autism spectrum disorder, Romantic partner, Friendship, Desires, Adolescents

## Abstract

Ideal friend and romantic partner characteristics related to self-perceived characteristics have been investigated in typically developing (TD) individuals, but not in individuals with autism spectrum disorder (ASD). Considering the autistic symptoms and challenges, investigating these concepts in autistic individuals is relevant. Given the lack of consensus, identity-first (“autistic person”) and person-first (“person with autism”) language are mixed throughout, to cover all preferences. This study explored (1) the association between self-perceived characteristics and desires in a friend/romantic partner, as well as (2) compare two groups (ASD and TD) in their desires for a friend/romantic partner. Two matched groups (ASD and TD) of 38 male adolescents (age 14–19 years) reported on the desire for nine characteristics (i.e., funny, popular, nice, cool, smart, trustworthy, good looking, similar interests, and being rich) in a friend/partner, and to what extent they felt they themselves possessed seven characteristics (i.e., funny, popular, nice, cool, smart, trustworthy, and good looking). Results showed both groups sought a friend and partner similar to themselves on intrinsic characteristics (e.g., trustworthiness), but less similar on extrinsic and social status characteristics (e.g., being less cool and popular). Particularly intrinsic characteristics, more than extrinsic and social status characteristics, were valued in both partners and friends, regardless of group. No significant differences were found between groups concerning to what extent characteristics were desired. Overall, adolescents with ASD desire similar characteristics as TD adolescents in their potential romantic partners and friends. There is some indication that the match between self-perception and desired characteristics is different.

## Introduction

Individuals with autism spectrum disorder (ASD) often have difficulties developing and maintaining friendships and romantic relationships (APA, 2013; Petrina et al., 2014).^1^ A long-held belief was that they preferred solitude and had no interest in social or intimate relationships (Ballan, 2012; Kanner, 1943). However, several studies have underlined that individuals with ASD have the desire for friendship and romantic relationships (Bauminger & Kasari, 2000; Calder et al., 2013; Cheak-Zamora et al., 2019; Hellemans et al., 2010; Henault, 2006; Stokes et al., 2007). Some research has found that, not a limited social motivation and desire, but rather difficultly to initiate and maintain romantic relationships exists for many adults with ASD (Strunz et al., 2017). Such issues may also exist in adolescents with ASD. Despite several successful evidence-based programs (e.g., Dekker et al., 2019; Laugeson et al., 2012; Visser et al., 2017) that aim to improve the social knowledge and skills that individuals with ASD have, it seems that in daily life, making and maintaining friendships or romantic relationships remain a challenge (Bauminger & Kasari, 2000; Church et al., 2000; Finke, 2016; May et al., 2017; Petrina et al., 2014; Rowley et al., 2012). One explanation for this is that, despite improved social communicational skills, there might be a mismatch between self-perceived (own) characteristics, preferences, attitudes, with desires about a potential friend or romantic partner in individuals with ASD (Finke, 2016).

The interplay between self-perceived own characteristics and desired characteristic in a friend or romantic partner is currently unknown in individuals with ASD. Research in typically developing (TD) individuals found that desired characteristics in a friend or a partner are related to self-perceived characteristics (i.e., the characteristics a person beliefs to possess) (Brown & Brown, 2015; Campbell et al., 2001; Kenrick et al., 1993; Sprecher & Regan, 2002). These studies showed that people who rate themselves highly as a potential friend or partner also have higher demands in what they desire in various relational partners (e.g., friendship or romantic relationship) (e.g., Regan et al., 2000).

Regardless of self-perceived characteristics, there are certain characteristics generally desired more in friendships and/or romantic relationships. Commonly listed desired friendship qualities mentioned by adolescents with ASD and TD adolescents entail; trustworthiness, kindness, companionship, and having similar interests (Locke et al., 2010; Petrina et al., 2014). In a qualitative study in adolescents and young adults with ASD ideal partners were described with characteristics like caring, kind, smart, romantic, and funny, whilst physical attractiveness was described as less important (Cheak-Zamora et al., 2019). In the TD population, three major components have been underlined as desirable in a romantic partner; i.e., warmth/trustworthiness, attractiveness/vitality, and status/resources (Fletcher et al., 2004). In TD adults, intrinsic characteristics (e.g., warmth, honesty, and intelligence) are desired to a greater degree than extrinsic characteristics (e.g., wealth and physical attractiveness) in romantic partners (Figueredo et al., 2006; Regan et al., 2000). Similarly, in TD adolescents extrinsic characteristics (e.g., physical attractiveness) were found to be more important for short-term sexual partners, and intrinsic characteristics (e.g., intelligence and humor) more so for long-term romantic partners (Regan & Joshi, 2003).

Friendship and romantic relationships have been studied in ASD populations; however, desired or idealized characteristics were usually not the focus. Most of the studies on friendships in individuals with ASD have investigated how individuals with ASD define friendship (i.e., what makes someone a friend; e.g., Petrina et al., 2014), whether they have friends (e.g., Rowley et al., 2012) and the quality of the friendships they have (e.g., Calder et al., 2013; Kuo et al., 2013; Locke et al., 2010). Research regarding romantic relationships in individuals with ASD has followed a similar pattern, with the focus on the existence of (e.g., Byers et al., 2013a; Fernandes et al., 2016; May et al., 2017; Strunz et al., 2017) and satisfaction with romantic relationships (e.g., Strunz et al., 2017). Although one study did find some characteristics to be mentioned as ideal (Cheak-Zamora et al., 2019), it remains unclear which characteristics are desired in their potential friends and romantic partners by adolescents with ASD, and if and how this may differ from TD adolescents.

The studies on friendships and romantic relationships in individuals with ASD have found that gender is related to the outcomes. Friendship studies have found that males with ASD have less social motivation (Sedgewick et al., 2016), less conflict experiences (Sedgewick et al., 2019b), less social skills (Head et al., 2014), different friendship understanding (Płatos & Pisula, 2021), and different activity patterns with friends (i.e., males more video gaming, females more conversations) (Kuo et al., 2013). Also, in romantic relationships gender has been linked to differences in sexual well-being in individuals with ASD. For example, males with ASD reported more dyadic arousability, greater desire, and greater sexual satisfaction (Byers et al., 2013b), more desire for sexual activity, less sexual anxiety and less sexual problems (Byers et al., 2013a), and less sexual orientation diversity (Dewinter et al., 2017; Turner et al., 2017). There is mixed evidence of how gender may be related to dyadic romantic or sexual relationships, with some finding men with ASD to have a greater engagement (Pecora et al., 2016) and others that males with ASD are less likely to be in a relationship (Turner et al., 2017). Given the gender differences, it is relevant to account for gender in research into friendships and romantic partners, as potentially it may translate to self-perceived characteristics and desired characteristics.

In addition, much of the research on friendships and romantic relationships was performed in primarily adult or clinical samples with ASD (May et al., 2017), while few have focused specifically on adolescents (e.g., Cheak-Zamora et al., 2019; Dekker et al., 2017; Dewinter et al., 2017). This despite the fact that in adolescence different characteristics may become more important in friendships; for example, loyalty and trustworthiness as intimate disclosure increase (Berndt, 1996). Similarly, romantic interests, dating, and social interaction with peers are increasingly important and are typical for this developmental period (Kuo et al., 2013; Regan & Joshi, 2003). In addition, interactions and experiences during this time allow for more complex emotions and romantic and social interactions later life. Such experiences also change and deepen romantic relationships, going from more friendship-like qualities to more bonding, intimacy, caring for one another and sexuality (Furman & Wehner, 1994). Furthermore, during adolescence, making and maintaining friendships and relationships becomes an individual task, rather than a task that is facilitated by parents or caregivers. In individuals with ASD particularly adolescence is a vulnerable period, as social problems typically worsen, as well as the emergence of feelings of loneliness and isolation (Locke et al., 2010). Findings in adolescent populations with ASD discuss negative experiences, such as anxiety, loneliness, limited opportunities to learn and practice social and romantic skills or to initiate relationships, and differences in sexual orientation (see Pecora et al., 2016 for elaborate discussion). However, these studies did not investigate desired characteristics in (potential) friends or romantic partners, especially in relation to self-perceived characteristics.

For many TD individuals, friendships are a steppingstone to romantic relationships (Fraley et al., 2013). Often the friendships, which become increasingly important during adolescence, serve as an opportunity to learn and practice social and relationship skills, as well as opportunities to meet potential partners (Glick & Rose, 2011; Seiffge-Krenke, 2003). There are studies that describe that a lack of friendships may decrease the possibility of practicing social/romantic skills (e.g., Strunz et al., 2017), which could escalate into inappropriate courting behavior (e.g., Stokes et al., 2007). In addition, qualities, which may be deemed valuable in a friend, are also often sought-after traits in a potential romantic partner (e.g., Sprecher & Regan, 2002), implying that desired characteristics in a friend may be at least to some extent related to desired characteristics in romantic partners. Lastly, congruence between friends (having similar characteristics) is important for a friendship to flourish (Bagwell & Schmidt, 2011). Therefore, knowing what adolescents with and without ASD would desire in their friends and/or romantic partners, also in relation to their own characteristics, may give insights into the trajectories of social and psychosexual development, for individuals with and without ASD. In turn, such knowledge could inform how support may be better tailored to assist in forming relationships and can be used to optimize existing evidence-based interventions, which could decrease loneliness and isolation.

Therefore, this study investigated the association of the desired characteristics in a friend and romantic partner with self-perceived (own) characteristics in adolescents with ASD, as well as in TD adolescents. This will provide information on whether adolescents look for someone similar (i.e., congruent) or different (i.e., complementing) to how they regard themselves. Given the known gender differences, as discussed above, in friendship and romantic relationships in individuals with ASD (e.g., Byers et al., 2013a, 2013b; Sedgewick et al., 2019b) and the limited number of females in our data set, we focused on adolescent males. Based on clinical observations, we hypothesized that male adolescents with ASD show a bigger discrepancy between their desired characteristics in a friend or romantic partner and their self-perceived characteristics than TD adolescents. As a first step, we compared which, and to what extent, characteristics adolescents with ASD desire in a romantic partner and friends, to TD adolescents. We hypothesized that adolescents with ASD desire different characteristics, and to a different extent (i.e., more or less), in their romantic partners and friends than TD adolescents. Second, we investigated the relation between self-perceived characteristics and friend and partner characteristics. We hypothesized that adolescents with ASD will be shown a larger discrepancy (more complementing) than TD adolescents (more congruent). Knowledge about desired characteristics, as well as the relation to self-perceived characteristics, may shed light on why individuals with ASD experience difficulties in forming and maintaining social relationships. The relevance of research on both the experiences and navigation of sexuality across the lifespan (priority ranking #3 out of 10) as well as how to support and promote sexual well-being (priority ranking #2 out of 10) has been underlined as relevant future research in this field by researchers, groups of people with ASD and other stakeholders (Dewinter et al, 2020).

## Method

### Participants and Procedure

Between 2011 and 2012, data for this study were collected from an ASD group and a TD group. As the ASD sample had a limited number of female adolescents, it was decided to only include the male adolescents in this study. This was done as gender can have a significant relation with partner preferences (e.g., Furnham, 2009; Hall, 2011) and the quality of a friendship (Kuo et al., 2013; Sedgewick et al., 2019a). Next, the two groups (ASD and TD) were matched on age with a maximum of one-month variation in age (i.e., 1-month fuzz in matching procedure). Matching on age was done because age may also influence which qualities are desired in a romantic partner and friend. Older participants include different and more information in their definition of romantic partners and friendship (Collins, 2003; Petrina et al., 2014), e.g., intimacy, mutual help/protection, and similarity in personality. In addition, matching, particularly in ASD research, is valuable to generate a more precise evaluation of potential differences with comparison groups (Burack et al., 2004). The sampling was set with a ratio of one adolescent with ASD to one TD adolescent. This ratio was chosen primarily to increase similarity between the two groups. As described in Stuart (2010) although it may reduce sample size, given that matched to the smallest sample the overall power may not be reduced much, and in addition, it can increase power due to reduced extrapolation and higher precision. In addition, in a larger matching ratio the final matching may have been poorer (i.e., matches may be less similar), leading to less comparability. By ensuring well-matched pairs in a relatively smaller sample, overall, the group is more comparable, and results can be interpreted with more certainty.

The ASD group (*N* = 38; ages 14 to 19) was selected from a larger clinical sample from the outpatient’s Department of Child and Adolescent Psychiatry/psychology of the Erasmus MC-Sophia Rotterdam, the Netherlands, who were participating in a follow-up study (de Bruin et al., 2007; Louwerse et al., 2015). For the current study only males with a best-estimate ASD diagnosis were included.

In the first study between July 2002 and September 2004, 503 children were referred for psychiatric evaluation. Of the 503 referrals, 234 children were eligible to be included in the follow-up study due to their social and/or communication problems at the time of the first study (see Eussen et al., 2015; Louwerse et al., 2015 for information on the procedure). Of the 234 children, for 104 (42.3%) children a best-estimate ASD diagnosis was indicated as based on the ADI-R and ADOS (see ASD diagnostic procedure below). Only those with a best-estimate ASD diagnosis received the questionnaire to participate in this part of the study. Of the 104 adolescents with a best-estimate ASD diagnosis, 58 (55.8%) returned the questionnaire. The group who did not return the questionnaire did not differ significantly from the group who did on age (*t*(77) = 0.68, *p* = 0.50), intelligence (*t*(59) = -1.43, *p* = 0.16), or gender (χ^2^(1, *n* = 79) = 0.46, *p* = 0.50). As only males were included (*n* = 49), the matching based on age resulted in a final ASD sample of 38 male adolescents. The mean age of the ASD group was 16.50 years (range 14.10–19.30, *SD* = 1.60) with a mean total IQ of 104.40 (range 71–135, *SD* = 14.5).

The TD group (*N* = 38; ages 14 to 19) was drawn from a Dutch general population study (*N* = 1710) (Evans et al., 2012; Louwerse et al., 2013; Tick et al., 2008) from which 326 individuals were eligible to participate based on their age (between 12 and 21 years old) (Evans et al., 2012). Of the 326 adolescents who were contacted, 113 (35%) returned the questionnaire. To ensure the TD group would be without autistic characteristics, we additionally excluded individuals if they had elevated autistic characteristics (*n* = 22) as assessed with the Autism Quotient (AQ; Baron-Cohen et al., 2001). This resulted in a sample of 91 adolescents (of which 38 male), who returned the questionnaire and were without autistic characteristics on the AQ. Those who did not return the questionnaire did not differ significantly from the group who did return the questionnaire on intelligence (*t*(118) = −0.66, *p* = 0.51), or gender (χ^2^(1, *n* = 131) = 1.96, *p* = 0.16). However, the group who did return the questionnaire was significantly younger (*M* = 16.12, *SD* = 1.47) than the group who did not (*M* = 16.73, *SD* = 1.77; *t*(129) = 2.03, *p* = 0.04). Matching of the TD adolescents with ASD group resulted in 38 TD adolescents to participate in the current study. The mean age of the TD sample (*n* = 38) was 16.35 years (range 14.1–19.3, *SD* = 1.66), and they had a mean total IQ of 103.61 (range 64–152, *SD* = 16.40).

The study was approved by the Medical Ethical Review Committee (MEC-2008–388). All adolescents gave informed consent and, where relevant due to their age, also the parents.

#### ASD Diagnostic Procedure

The best-estimate clinical diagnosis of ASD was obtained by means of the Autism Diagnostic Interview Revised (ADI-R; Rutter et al., 2003) and the Autism Diagnostic Observation Schedule (ADOS; Lord et al., 2000). Examiners who completed the research-training and had achieved sufficient reliability for administration and coding administered the ADI-R and ADOS. Module 4 of the ADOS was primarily used, based on age and language capability of the sample, although for six participants the ADOS module 3 was used. The examiner of the ADI-R and the examiner of the ADOS reviewed DSM-IV-TR criteria of ASD (i.e., Pervasive Developmental Disorder) and together they obtained a consensus diagnosis (Falkmer et al., 2013).

### Measures

#### Characteristics

In the current study, we used a part of the Teen Transition Inventory (TTI; Dekker et al., 2017) which was developed using feedback of adolescents with ASD on wording and content. Seven characteristics were rated by the participants three times, first, to what level they possessed the characteristic, second, to what level they desired this characteristic in a partner, and third, to what level they desired this characteristic in a friend. The seven characteristics were: funny, popular, nice, cool, smart, trustworthy, and good looking. Two additional characteristics were part of the list of characteristics for a friend and partner, i.e., similar interests and being rich. All characteristics were rated on a 3-point rating scale (“Not at all,” “Somewhat,” and “Definitely”).

The wording in the TTI (Dekker et al., 2017) was aimed to be as concrete and non-ambiguous as possible, to better suit the ASD population as well as the age of the participants. The characteristics in the current study were based on terms used in the previous research in TD young adults (e.g., Sprecher & Regan, 2002). The characteristics can be roughly divided into three categories: intrinsic characteristics (i.e., trustworthy, nice, funny, smart, and similar interests), extrinsic characteristics (i.e., good looking), and social status (i.e., popular, cool, rich). The latter two categories are generally not used in descriptions of high-quality friendship, but have been found to be more relevant for partner choice, as well as for adolescents as social status is increasingly important then (Caravita et al., 2009; Sprecher & Regan, 2002).

#### Physical Development

For physical development, we used the Tanner stages, a staging system which uses schematic drawings of secondary sexual characteristics divided into five standard stages (Marshall & Tanner, 1969, 1970). The adolescent indicated on the gender appropriate sketches which stage resembled the physical appearance of himself/herself most. The ratings have been used widely and shown good reliability and validity (Dorn et al., 1990).

### Analyses

#### Descriptive Statistics

As only males were selected and the two groups are matched on age, no significant differences were expected on these descriptive characteristics. To ensure this, and for other descriptive characteristics, we investigated whether the two groups differed in age, self-reported physical development (i.e., Tanner stages), and intelligence. Furthermore, for the ASD group we analyzed the severity of their ASD by means of the calibrated severity score (Gotham et al., 2009; Hus et al., 2014) on the ADOS (Lord et al., 2000) as well as their score on the ADI–R (Lord et al., 1994) (see section ASD Diagnostic Procedure for more information). In addition, we investigated how many adolescents in our sample claimed to have experience with friendship and romantic relationships, as well as if they thought they would make a good friend or partner. Finally, for each group (ASD and TD) we ranked the characteristics of how they view themselves from most present to least present, as well as in a friend and a partner from most desired to least desired.

#### Group Differences Friend/Partner Characteristics

To investigate differences in desired characteristics in a friend between the adolescents with ASD and the TD adolescents, we ran chi-square tests to check whether the groups (ASD vs. TD) differed significantly in how much they desire each characteristic in a friend. Similar analyses were performed to examine desired characteristics in a partner.

#### Relation Self-Perception to Friend or Partner Characteristics

Our main goal was to investigate whether there would be a significant relation between self-perceived level of having characteristics and the level to which these characteristics are desired in a friend and a partner. For this purpose, we ran Wilcoxon signed-rank tests for both groups (ASD and TD) comparing self to friend characteristics and self to partner characteristics.

## Results

### Sample Descriptives

There were no significant differences in age, intelligence, and self-reported physical development (Tanner stage) between the two groups (see Table 1). Furthermore, the majority of the adolescents in both groups reported that they currently had at least one meaningful friendship. About two-third in both groups reported they have dated someone, which means they also did not differ significantly in this respect. Interestingly, there was a significant difference in how the adolescents consider themselves in terms of being a good friend or a good romantic partner. Adolescents with ASD considered themselves to be a lesser friend (*M* = 2.60, *SD* = 0.50), than their TD peers (*M* = 2.82, *SD* = 0.46; *t*(73) = −2.00, *p* = 0.048), as well as a significantly lesser partner (ASD: *M* = 2.14, *SD* = 0.72; TD: *M* = 2.50, *SD* = 0.69; *t*(72) = −2.20, *p* = 0.03).

**Table 1:** Characteristics of participants

	ASD				TD				*p*
	N (%)	Mean	SD	Range	N (%)	Mean	SD	Range	
Age	38 (100%)	16.50	1.60	14.1–19.3	38 (100%)	16.35	1.66	14.1–19.3	.68
Intelligence	38 (100%)	104.4	14.5	71–135	38 (100%)	103.61	16.40	64–152	.84
Tanner stage	38 (100%)	3.97	.96	1–5	38 (100%)	4.24	.80	2–5	.20
ADI-R Diagnostic	38 (100%)	37.11	11.16	4–59					
ADI-R Current	37 (97.4%)	18.92	6.61	9–36					
ADOS CSS	37 (97.4%)	5.87	2.32	1–10					

	N (%)	Yes N (%)	Somewhat N (%)	No N (%)	N (%)	Yes N (%)	Somewhat N (%)	No N (%)	*p*
Meaningful friendship—self	36 (94.7%)	31 (86.1)	–	5 (13.9)	37 (97.4%)	35 (94.6)	–	2 (5.4)	.20
Have dated someone	38 (100%)	25 (65.8)	–	13 (34.2)	38 (100%)	26 (68.4)	–	12 (31.6)	.81
Need for a friend—self	37 (97.4%)	15 (40.5)	15 (40.5)	7 (18.9)	36 (94.7%)	13 (36.1)	12 (33.3)	11 (30.6)	.51
Think they are a good friend	37 (97.4%)	22 (59.5)	15 (40.5)	0 (0)	38 (100%)	32 (84.2)	5 (13.2)	1 (2.6)	.048
Think they are a good romantic partner	36 (94.7%)	12 (33.3)	17 (47.2)	7 (19.4)	38 (100%)	23 (60.5)	11 (28.9)	4 (10.5)	.031

*ASD* = autism spectrum disorder, *TD* = typically developing. *ADI-R* = autism diagnostic interview—revised. *ADOS CSS* = autism diagnostic observation schedule calibrated severity score

*One participant was in a clinical evaluation process at another facility, for this participant only the diagnostic ADI-R score was available

Table 2 shows the rankings of the different characteristics per group, regarding what the adolescents desire in a partner and in a friend, as well as how the adolescents view themselves. The results showed that for desired characteristics in partners, the top four of the ranking was the same in the ASD group and the TD group (with some variation in percentages), but the other five characteristics are ranked slightly different. Regarding desired characteristics in a friendship, it stood out that place one and two in the ranking are switched when comparing the ASD and TD groups, but primarily there was a consensus between the two groups in terms of ranking. As for the characteristics of themselves, the ranking was the same for the first four places, although the number of adolescents who claim this characteristic ‘suits them very much or often’ did differ (i.e., lower percentages in the ASD group).

**Table 2:** Ranking of characteristics per group regarding desires in friends, partners, and of self

		Friendship		Median	Partner		Median	Self		Median
ASD	1	Trustworthy	91.9%	Very or often true	Trustworthy	86.5%	Very or often true	Trustworthy	78.4%	Very or often true
	2	Nice	88.9%	Very or often true	Nice	78.4%	Very or often true	Nice	56.8%	Very or often true
	3	Funny	36.1%	Somewhat or sometimes true	Funny	32.4%	Somewhat or sometimes true	Smart	54.1%	Very or often true
	4	Same interests	24.3%	Somewhat or sometimes true	Good looking	32.4%	Somewhat or sometimes true	Funny	43.2%	Somewhat or sometimes true
	5	Good looking	7.9%	Not at all true	Same interests	21.6%	Somewhat or sometimes true	Popular	23.7%	Somewhat or sometimes true
	6	Cool	2.8%	Not at all true	Smart	8.8%	Somewhat or sometimes true	Good looking	18.9%	Somewhat or sometimes true
	7	Smart	2.7%	Somewhat or sometimes true	Cool	5.4%	Not at all true	Cool	13.5%	Somewhat or sometimes true
	8	Popular	2.7%	Not at all true	Popular	5.4%	Not at all true			
	9	Rich	0%	Not at all true	Rich	2.7%	Not at all true			

		Friendship			Partner			Self		
TD	1	Nice	89.2%	Very or often true	Trustworthy	86.8%	Very or often true	Trustworthy	94.4%	Very or often true
	2	Trustworthy	83.8%	Very or often true	Nice	84.2%	Very or often true	Nice	78.9%	Very or often true
	3	Funny	16.7%	Somewhat or sometimes true	Funny	39.5%	Somewhat or sometimes true	Smart	65.8%	Very or often true
	4	Same interests	13.5%	Somewhat or sometimes true	Good looking	36.8%	Somewhat or sometimes true	Funny	50%	Very or often true
	5	Smart	2.7%	Somewhat or sometimes true	Smart	26.3%	Somewhat or sometimes true	Good looking	29.7%	Somewhat or sometimes true
	6	Good looking	0%	Not at all true	Same interests	18.4%	Somewhat or sometimes true	Cool	21.1%	Somewhat or sometimes true
	7	Cool	0%	Not at all true	Popular	0%	Not at all true	Popular	10.8%	Somewhat or sometimes true
	8	Popular	0%	Not at all true	Rich	0%	Not at all true			
	9	Rich	0%	Not at all true	Cool	0%	Not at all true			

Percentages reflect the answer ‘Very or often true’. Ranking based on all answers (including ‘Somewhat’ and ‘Not at all’). ASD = autism spectrum disorder; TD = typically developing

### Group Differences Friend Characteristics

For friend characteristics, we ran Chi-square tests to investigate whether the scoring per characteristic differed significantly between the two groups. As can be seen in Table 3, none of the scores significantly differed between the ASD and TD groups. This indicates that for friends, both groups desired each characteristic to a similar level.

**Table 3:** Group comparison of desired friend characteristics

	Very % (*n*)	Somewhat % (*n*)	Not at all % *(n)*	χ^2^	df	*p*
Trustworthy				2.28	2	.32
ASD	91.9 (34)	8.1 (3)	0 (0)			
TD	83.8 (31)	10.8 (4)	5.4 (2)			
Nice				1.15	2	.56
ASD	88.9 (32)	8.3 (3)	2.8 (1)			
TD	89.2 (33)	10.8 (4)	0 (0)			
Funny				4.33	2	.12
ASD	36.1 (13)	41.7 (15)	22.2 (8)			
TD	16.7 (6)	63.9 (23)	19.4 (7)			
Same interests				1.52	2	.47
ASD	24.3 (9)	51.4 (19)	24.3 (9)			
TD	13.5 (5)	62.2 (23)	24.3 (9)			
Good looking				3.08	2	.21
ASD	8.1 (3)	37.8 (14)	54.1 (20)			
TD	0 (0)	38.9 (14)	61.1 (22)			
Cool				2.74	2	.25
ASD	2.8 (1)	44.4 (16)	52.8 (19)			
TD	0 (0)	30.6 (11)	69.4 (25)			
Smart				1.19	2	.55
ASD	2.7 (1)	78.4 (29)	18.9 (7)			
TD	2.7 (1)	67.6 (25)	29.7 (11)			
Popular				3.65	2	.16
ASD	2.7 (1)	32.4 (12)	64.9 (24)			
TD	0 (0)	16.7 (6)	83.3 (30)			
Rich				.13	2	.72
ASD	0 (0)	13.5 (5)	86.5 (32)			
TD	0 (0)	10.8 (4)	89.2 (33)			

*ASD* = autism spectrum disorder; *TD* = typically developing

### Group Differences Partner Characteristics

Similarly, to test whether the ranking of partner characteristics differed between the two groups, we ran Chi-square tests. As can be seen in Table 4, none of the scorings significantly differed between the ASD and TD groups. This means that for each partner characteristic both groups desired it to a similar level.

**Table 4:** Group comparison of desired partner characteristics

	Very % (*n*)	Somewhat % (*n*)	Not at all % (*n*)	χ^2^	df	*p*
Trustworthy				1.11	2	.57
ASD	86.5 (32)	13.5 (5)	0 (0)			
TD	86.8 (33)	10.5 (4)	2.6 (1)			
Nice				.47	2	.79
ASD	78.4 (29)	18.9 (7)	2.7 (1)			
TD	84.2 (32)	13.2 (5)	2.6 (1)			
Funny				1.35	2	.51
ASD	32.4 (12)	51.4 (19)	16.2 (6)			
TD	39.5 (15)	52.6 (20)	7.9 (3)			
Same interests				1.21	2	.55
ASD	21.6 (8)	54.1 (20)	24.3 (9)			
TD	18.4 (7)	65.8 (25)	15.8 (6)			
Good looking				1.24	2	.54
ASD	32.4 (12)	51.4 (19)	16.2 (6)			
TD	36.8 (14)	55.3 (21)	7.9 (3)			
Cool				3.71	2	.16
ASD	5.4 (2)	32.4 (12)	62.2 (23)			
TD	0 (0)	21.1 (8)	78.9 (30)			
Smart				4.01	2	.14
ASD	8.8 (3)	82.4 (28)	8.8 (3)			
TD	26.3 (10)	63.2 (24)	10.5 (4)			
Popular				2.21	2	.33
ASD	5.4(2)	24.3 (9)	70.3 (26)			
TD	0 (0)	28.9 (11)	71.1 (27)			
Rich				1.20	2	.55
ASD	2.7 (1)	27.0 (10)	70.3 (26)			
TD	0 (0)	23.7 (9)	76.3 (29)			

*ASD* = autism spectrum disorder; *TD* = typically developing

### Relation Self-Perception to Friend or Partner Characteristics

Lastly, we investigated whether adolescents seek a partner or friend who is like themselves (i.e., congruent) or dissimilar to themselves (complementary). Our results showed that for both the ASD and TD groups, friends were desired who are slightly less like themselves (i.e., more complementary with self) than partners (i.e., more congruent with self).

With regard to friends, the Wilcoxon signed-rank tests indicated that five of the seven characteristics were ranked significantly different in the ASD group when comparing what was desired in a friend and how they viewed themselves. Specifically, the characteristics on which adolescents with ASD rank themselves significantly different compared to what they would desire in a friend were: being popular (*z* = −4.08; *p* < 0.001), being nice (*z* = −2.45; *p* = 0.01), being cool (*z* = −2.11; *p* = 0.04), being smart (*z* = −4.33; *p* < 0.001) and being good looking (*z* = −3.52; *p* < 0.001). Adolescents with ASD desired a best friend who would be less popular, less cool, less smart, and less good looking but nicer than that they regarded themselves. The TD group showed similar results, with also five characteristics ranked significantly different. In the TD group, a friend was desired who is significantly less funny (*z* = −3.28; *p* < 0.01), less popular (*z* = 5.14; p < 0.001), less cool (*z* = −4.63; *p* < 0.001), less smart (*z* = −5.06; *p* < 0.001), and less good looking (*z* = −4.49; *p* < 0.001) than how they ranked themselves. In contrast to the ASD group, there was no significant difference regarding the characteristic being nice (*z* = −1.63; *p* = 0.10), but there was a significant difference on the characteristic funny (desire a less funny friend). The only characteristic which showed no significant difference in both groups was ‘trustworthy’. Thus, both ASD and TD adolescents desire a friend who was similar in terms of trustworthiness compared to how they rated themselves.

With regard to a partner, the Wilcoxon signed-rank tests showed that for four of the seven characteristics, adolescents with ASD rank themselves differently from what they would seek in a partner. Adolescents with ASD desired a partner who is less popular (*z* = −4.15; *p* < 0.001), less cool (*z* = −2.43; *p* = 0.02); less smart (*z* = −3.79; *p* < 0.001), but nicer (*z* = −1.97; *p* = 0.05) than they would rate themselves on these variables. In the TD group, three characteristics were rated significantly different when comparing self-image to what is desired in a partner. TD adolescents desired a partner who is less popular (*z* = −4.84; *p* < 0.001), less cool (*z* = −4.72; *p* < 0.001), and less smart (*z* = −3.51; *p* < 0.001) than that they regarded themselves on these variables. In contrast to the ASD adolescents, no significant difference was found regarding the characteristic nice. No significant differences were found on the other variables, i.e., trustworthiness, funny, and good looking.

The results show that for both friends and partners the adolescents with ASD and TD had similar desires in relation to their own characteristics. The only differences found were on the characteristic nice and funny. For the characteristic nice only, a significant difference in ranking was found for the adolescents with ASD, where they desired someone who is nicer compared to how they rated themselves (both in a partner and a friend). For the characteristic funny only for a friend a significant difference was found in ranking in the group of TD adolescents. TD adolescents desired a friend, but not a partner, who is less funny than how they rated themselves.

When including all the males in both samples (*n* = 49 ASD and *n* = 38 TD), overall similar results were found.

## Discussion

In the past few decades, the empirical and academic interest in romantic relationships and friendships in adolescents with ASD has increased (e.g., Bauminger & Kasari, 2000; Dewinter et al., 2020; Henault, 2006; Pecora et al., 2016). However, it remained unclear which characteristics individuals with ASD desire in a partner or friend, and how these relate to characteristics the adolescents think they themselves possess. The current study investigated desired characteristics in friends and romantic partners, in relation to self-perceived own characteristics, comparing adolescents with and without ASD, in order to examine to what extent, they desire a similar (i.e., congruent) or dissimilar (i.e., complementing) partner or friend to how they perceive themselves. Our study revealed that adolescents with ASD and TD have similar desires regarding which, and how much, they seek certain characteristics in both their partners and friends. Furthermore, it showed that both adolescents with ASD and TD desire a friend and partner who is congruent in some ways (e.g., intrinsic characteristics), but also complementing (e.g., social status characteristics) to their own characteristics.

More specifically, the results showed that adolescents with ASD have similar desires in their partners and friends regarding nine characteristics (i.e., funny, popular, nice, cool, smart, trustworthy, good looking, similar interests, and being rich), when compared to TD adolescents. Also, it showed that particularly intrinsic characteristics (i.e., trustworthy, nice, and funny) are valued more than extrinsic characteristics (i.e., good looking) and social status characteristics (e.g., popular, and rich). The lack of difference between the two groups contradicts our hypothesis that possibly adolescents with ASD desire different characteristics in their relationships. A possible explanation may be that society has an influence on which characteristics are considered to be more desirable than others. This may be subconsciously transferred by parents, caregivers, and peers, but also more explicitly in psycho-educational material or training programs regarding intimate relationships, which may internalize to rules that adolescents with ASD conform to. As rule-breaking is experienced differently and more distressing in individuals with ASD compared to TD adolescents (Bolling et al., 2011), this may explain why they report to desire similar characteristics as their TD peers. More research would be necessary to determine whether indeed desired characteristics are influenced by societal influences and/or opinions of authority figures.

Our main research question was to explore whether adolescents seek someone similar or dissimilar to themselves. Our results showed that both adolescents with ASD and TD adolescents reported to seek people (both partner and friend) who are relatively similar in terms of intrinsic characteristics, but less similar in terms of social status characteristics (i.e., less strong need to be popular, and cool). One difference that stands out is that adolescents with ASD, but not TD adolescents, seek someone who is nicer than themselves, both in a partner and a friend. Several possible explanations for this difference can be found in the adolescents with ASD themselves. Firstly, and interestingly, the difference seems to be caused by the lower score on self-rating rather than a difference in what is desired in friend/partner. This may be indicative of relatively low self-esteem in the adolescents with ASD, especially in terms of their own niceness. This is in line with other research, which has found that children and adolescents with ASD have significantly lower self-esteem than TD children and adolescents (Dekker et al., 2017; McCauley et al., 2019; van der Cruijsen & Boyer, 2021). In addition, the current study showed that there was a significant difference in how the adolescents viewed themselves in romantic relationships and friendships: significantly less adolescents with ASD felt confident that they would be a good partner as well as a good friend, than the matched TD adolescents. A possible explanation may be that particularly cognitively able adolescents with ASD—of which our sample existed—are aware of their social difficulties, which may lead them to think they are less suitable partners and friends for others. Although this requires more research, it is in line with previous research, which has shown that particularly the awareness of the social difficulties can have negative results (Locke et al., 2010; Sterling et al., 2008). Another explanation could be earlier experiences with peer rejection, which is common for many with ASD (Carrington & Graham, 2001; Symes & Humphrey, 2010). Possibly, previous peer rejection may have caused individuals with ASD to assume they will not be good partners or friends. This may also putatively explain the difficulties individuals with ASD have with developing and maintaining relationships, as low self-esteem may lead to less optimal outcomes (e.g., Takizawa et al., 2014) and correlates significantly with loneliness (Mazurek, 2014) and lower friendship qualities (Bauminger et al., 2004). Future research could investigate self-esteem in relation to self-perceived characteristics and desired characteristics in populations with ASD.

Interestingly, the results also show that adolescents with ASD seek someone who is closer to their own characteristics than TD adolescents, particularly in friends. Research in TD individuals found that the importance of ideal standards for a partner is calibrated to match one’s self-perception on the same dimensions (Brown & Brown, 2015; Campbell et al., 2001; Kenrick et al., 1993; Sprecher & Regan, 2002). Although previous research has suggested that individuals with ASD may be less capable of self-reflection and insight into their own functioning (Cederlund et al., 2010), our results suggest that cognitively able adolescents with ASD may in fact adapt their desires for partners and friends more to their own capabilities in those domains than TD adolescents. This could indicate a certain level of self-reflection and flexibility in real-life, alternatively, individuals with ASD may simply seek individuals who are ‘less’ desirable and therefore closer match their own lower self-image.

There are several noteworthy limitations to the current study. Here we discuss the four most prominent limitations. First, a limited number of characteristics were investigated, and it would be valuable to include more characteristics and possibly allow adolescents to add characteristics they desire. For example, the characteristic “dependable” has been found in the previous research to be important (Figueredo et al., 2006), but was not included in our list. Second, each characteristic was rated on a 3-point scale, which may have allowed insufficiently for variance. Future research could use a wider range. Third, our sample was limited, with 38 participants per group and only males. Given the differences in social and romantic functioning in females with ASD, it would be valuable to investigate desired characteristics in both females and males with ASD as well. Last, the ASD group consisted of predominantly average to high IQ, which decreases the generalizability of our findings to the more heterogeneous group of adolescents with ASD. Larger more diverse groups could provide more insight into which characteristics are desired by adolescents with ASD.

Despite the limitations, the current study adds new insights to the growing literature on the topic of psychosocial and psychosexual functioning in individuals with ASD. First, the current study is the first to investigate self-perceived characteristics in relation to desired characteristics in individuals with ASD and directly compare TD adolescents with adolescents with ASD. Second, self-report is still not commonly used in research into psychosexual functioning in adolescents with ASD (Fernandes et al., 2016). Although self-reflection or self-evaluation within children and adolescents with ASD has been challenged over time with respect to the reliability (e.g., Cederlund et al., 2010) and potential overestimation (Furlano et al., 2015), more recent studies have underlined the value of self-report and the insight to the personal experience of children and adolescents with ASD (e.g., Keith et al., 2019; Stokes et al., 2017). For example, the study of Cage et al. (2016) showed that adolescents (age 12 to 15) with ASD do have a self-concept and can report on it. In a study investigating the relation between survey information and autonomic measures (Keith et al., 2019), a high correlation between the two types was found, implying that self-report can provide unique insights into the experience of adolescents with ASD themselves. Even more so, a study on quality of life (Stokes et al., 2017) highlighted that parents are perhaps poor proxies of the experience of children with ASD (ages 6 to 20). Although individuals with ASD may over-estimate their competencies (e.g., Furlano et al., 2015), self-report does provide insights into how they think they are doing. Potentially, adapting self-perception to more reflect reality may be something useful in also seeking friends and romantic partners that better match this. By asking the adolescents themselves what they desire in a friend/partner, more insight is gained into their experiences, rather than the opinion of others. As the adolescents must establish and maintain relationships more and more on their own—as opposed to with help of parents or caregivers—information about their personal desires and experiences can better inform how parents, caregivers, and healthcare professionals may be able to support the adolescents. Third, by making use of a TD comparison group as well as using the same measurement in both groups, our results allow for a direct comparison of adolescents with ASD to a TD sample. Thereby, we can more clearly indicate whether and how adolescents with ASD differ from TD adolescents. As we matched our sample, our results cannot be caused by factors such as gender, age, and intelligence. Fourth, adolescence is the period in which increasingly time is spent with other gender peers and romantic partners (Collins et al., 2009). Also, previous research has shown that there is no significant difference in age of onset regarding, for example, sexual activity between adolescents with ASD and TD adolescents (Dekker et al., 2017). Therefore, investigating preferences for characteristics of friends and romantic partners during this period may shed more light on potential differences in the developmental paths of relationships between adolescents with ASD and their TD peers.

For future research, it may be beneficial to also include qualitative data, to go more into depth on how the adolescents would define certain characteristics and why they find them important, and how it may relate to their self-perception. Furthermore, it would be interesting to also receive information from individuals who are identified as friend or partner as well as relationship satisfaction, to investigate whether indeed the desired characteristics are in line with the partner’s or friend’s actual characteristics. This could perhaps also shed light on if the stated desires match actual characteristics of a friend/partner or if the reported desires are what they think is expected. In addition, investigating desired characteristics and self-perceived characteristics in relationship to self-esteem and in a more diverse population can further elucidate the interplay in individuals with ASD and how it may relate to social and romantic outcomes. Lastly, it may be valuable to include larger samples, to also allow for more complex analyses, for example a form of multivariate testing with profiles, and to be able to include other influential variables, such as physical maturation.

A possible clinical implication of our findings could be that adolescents with ASD may need different support in terms of establishing and maintaining successful long-term interpersonal relationships, a topic which was previously highlighted as an important research focus (Dewinter et al., 2020). Increasing their self-esteem, or at least nuancing their self-image, may benefit adolescents with ASD to find and maintain romantic and friendly relationships. In addition, it could be beneficial to train adolescents with ASD in self-awareness and self-presentation, so if they desire, they can be more desirable for others as a potential partner or friend. Another clinical implication could be increasing the willingness of adolescents with ASD to allow trade-offs between their desires and actual partners/friends. Clinicians, but also parents and other professionals, could perhaps work on the tolerance of individuals with ASD for imperfectness in self, partners and friends, which could lead to more long-lasting mental health and relationships. Although more research is required to see whether, in situations in which personal relationships are established, indeed self-esteem/image, self-awareness/presentation, or flexibility is a problem for adolescents with ASD, it could be valuable to promote these things in support programs for adolescents with ASD, so they may have a better chance at establishing and maintaining personal relationships.

Although beyond the scope of our research, two potential mechanisms may be related to difficulties with high-quality friendship and relationship outcomes and our current findings. First, lower self-esteem and less strategic self-presentation may also lead to less personal relationships. The characteristics sought after by both TD adolescents and adolescents with ASD are elements which individuals with ASD may struggle to offer or present (e.g., being nice). Some research has shown that children with ASD are less strategic and convincing in their self-presentation when seeking personal gain (Begeer et al., 2008). Second, difficulty with flexibility may be another reason why adolescents with ASD struggle with establishing and maintaining personal relationships. Often actual partners or friends will not show all the desired qualities, as there is a trade-off in real relationships (Figueredo et al., 2006; Varella Valentova et al., 2016). Speculatively, in real life adolescents with ASD allow less of a trade-off between what is desired and what an actual friend/partner has as characteristics than TD adolescents. This may lead to a more unattainable combination of characteristics (e.g., an unrealistic combination of characteristics), desired in their romantic partners and friends than TD individuals. Clearly, more research is needed to examine these speculations.

To summarize, our results indicate that in fact adolescents with ASD desire similar characteristics to a similar level as TD adolescents in their partners and friends. And, also comparable to TD adolescents, adolescents with ASD seek a partner and friend who is similar in most intrinsic characteristics, but less similar in other characteristics (i.e., social status and extrinsic characteristics) to themselves. As earlier research has shown that in fact individuals with ASD have a desire for romantic relationships and friendships, our research extends this work by showing that they also desire the same characteristics in their partners and friends as well as the level of congruency they seek with friend/partner as TD adolescents. Although a valuable first step, more research is required to clarify and replicate our findings to translate the current findings into support for adolescents with ASD in successfully navigating relations and sexuality throughout the lifespan. Knowledge on self-perception and desired characteristics may help to better tailor support programs aimed to improve romantic and friendship outcomes, for example by increasing awareness of own characteristics (i.e., better self-awareness), by improving self-presentation to peers, or by aiding in finding a better match in a potential partner or friend. For future research, it would be valuable to investigate more characteristics in a larger and more diverse sample, preferably including self-esteem as well as self-evaluation.

## Data Availability

Data and material are not publicly available.

## Footnotes

1 Given the lack of consensus on identity-first or person-first language (e.g., Kenny et al., 2016), a mix is used throughout this paper.
